# *Trichogramma ostriniae* Is More Effective Than *Trichogramma dendrolimi* As a Biocontrol Agent of the Asian Corn Borer, *Ostrinia furnacalis*

**DOI:** 10.3390/insects13010070

**Published:** 2022-01-08

**Authors:** Yu Wang, Yang-Yang Hou, Giovanni Benelli, Nicolas Desneux, Asad Ali, Lian-Sheng Zang

**Affiliations:** 1 Engineering Research Center of Natural Enemies, Institute of Biological Control, Jilin Agricultural University, Changchun 130118, China; WangY19940504@163.com (Y.W.); tiantian1214418@163.com (Y.-Y.H.); 2 Department of Agriculture, Food and Environment, University of Pisa, via del Borghetto 80, 56124 Pisa, Italy; giovanni.benelli@unipi.it; 3 Université Côte d’Azur, INRAE, CNRS, UMR ISA, 06000 Nice, France; 4 Department of Entomology, Abdul Wali Khan University, Mardan 23200, Pakistan; a.ali07@alumni.imperial.ac.uk; 5 Key Laboratory of Green Pesticide and Agricultural Bioengineering, Guizhou University, Guiyang 550025, China

**Keywords:** biological control, Crambidae, egg age, host age, oophagous parasitoid, Trichogrammatidae

## Abstract

**Simple Summary:**

The performance of two egg parasitoids *T. dendrolimi* and *T. ostriniae* were compared on Asian corn borer (ACB) *Ostrinia furnacalis* eggs of different ages under choice and no-choice conditions. Both parasitoid species showed significant preferences in parasitizing ACB eggs of different ages. Younger ACB eggs (0–8-h-old) showed high suitability while eggs older than 8 h were not suitable for *T. dendrolimi*. The age of ACB eggs did not affect the biological parameters of *T. ostriniae*. Overall, our results highlighted the *T. ostriniae* species as the best candidate biocontrol agent for managing ACB populations.

**Abstract:**

The Asian corn borer (ACB), *Ostrinia furnicalis*, is a serious corn pest in south-east Asia, causing huge economic losses every year. *Trichogramma dendrolimi* and *Trichogramma ostriniae*, two egg parasitoids, have previously been identified as key biological control agents. To determine the age impact of ACB eggs on their effective biocontrol potential, herein we compared the biological parameters (i.e., number of parasitized eggs, emergence, developmental time, and sex ratio) of both parasitoids on ACB eggs of various ages (i.e., 0–4, 4–8, 8–12, 12–16, 16–24, 24–36, and 36–48 h old), respectively. Our results showed that the age of ACB eggs had a significant impact on the parasitization activity of *T. dendrolimi* in both choice and no-choice conditions. *Trichogramma dendrolimi* preferred to parasitize 0–8-h-old ACB eggs, and its parasitization dramatically declined on ACB eggs older than 8 h under choice and no-choice conditions. On the other hand, *T. ostriniae* showed high preference to parasitize all tested ACB egg ages. The age of ACB eggs had no significant impact on the parasitization of *T. ostriniae* under choice and no-choice conditions. Furthermore, the female progeny of *T. dendrolimi* decreased as the age of ACB increased, while no differences were found in female progeny of *T. ostriniae*. *Trichogramma ostriniae* also developed faster on each ACB egg age group in comparison with *T. dendrolimi*. Overall, the age of ACB eggs had a significant impact on *T. dendrolimi* performance, leading us to conclude that *T. ostriniae* is more effective than *T. dendrolimi* as a biocontrol agent of the ACB.

## 1. Introduction

The Asian corn borer (ACB), *Ostrinia furnicalis* Guenée 1854 (Lepidoptera: Crambidae), the most destructive maize pest, is distributed throughout south-east Asia, especially in China, where it causes up to 30% yield losses yearly [1,2]. Recently, ACB infestation significantly increased in maize-producing regions, due to changes in climate and farming systems (i.e., increased plantation density and tillage method of straw returning). As a result, insecticide application for managing ACB has increased. However, the indiscriminate and frequent overuse of insecticides leads to serious side effects, such as pest resistance and resurgence, as well as hormesis [3,4,5,6], coupled with a negative impact on beneficial arthropods and human health [7,8,9,10]. To avoid these issues, it is essential to establish effective and environmentally sustainable ACB management strategies in maize fields [11]. Among the pest management strategies currently available, the use of *Trichogramma* parasitoids as ACB biocontrol agents has been outlined [12].

*Trichogramma* (Hymenoptera: Trichogrammatidae) is an important genus of biological control agents, comprising many species that are currently used for managing various moth pests, with significant ecological and economic advantages [12,13,14,15,16,17,18]. The releases of *Trichogramma* parasitoids for the management of moth pests, especially ACB, are some of the most important measures in maize Integrated Pest Management (IPM) in China. Notably, *Trichogramma*-treated corn area in northeast China has increased from 0.6 to 5.5 million ha between 2005 and 2015 [12,19,20].

*Trichogramma dendrolimi* Matsumura and *Trichogramma ostriniae* (Pang & Chen) have been reported as the best potential biological control agents against ACB among the available *Trichogramma* species [12,21]. In Northeastern China, inundative releases of *T. dendrolimi* alone (225,000 parasitoids ha^−1^) have covered an area of 2.3 million ha yearly since 2012 [12]. In addition, inundative releases of *T. ostriniae* (75,000–120,000 parasitoids ha^−1^) resulted in >90% parasitization of ACB eggs [12,22]. The area-wide release of these *Trichogramma* parasitoids has been continuously expanding, and their value as an effective tool for sustainable ACB management has been recognized [23,24]. However, despite the results obtained with these parasitoid species, there are unsolved issues on the selection of best *Trichogramma* species to use [12].

To ensure effectiveness of biological control programs, it is key to assess the suitability of targeted pest(s) for selected parasitoid(s) [25,26,27,28,29]. The sensitivity of *T. dendrolimi* to the age of ACB eggs was found to be a key factor, affecting its parasitization ability [30]. On the other hand, *T. ostriniae* is widely regarded as the most effective ACB egg biocontrol agent [12]. However, the age effect of ACB egg on the biological parameters of *T. ostriniae* in comparison to *T. dendrolimi* is still unknown.

Therefore, in this study, the parasitization ability of *T. dendrolimi* and *T. ostriniae* on ACB eggs of various ages was investigated under choice and no-choice conditions. Furthermore, we assessed the sex ratio, developmental time, and emergence rate of both *Trichogramma* species on ACB eggs of various ages.

## 2. Materials and Methods

### 2.1. Parasitoids

The parasitoids *T. dendrolimi* and *T. ostriniae* were initially collected from parasitized eggs of the rice stem borer, *Chilo suppressalis* (Lepidoptera: Pyralidae), in paddy fields in Changchun, Jilin province, China (43.89° N, 125.32° E). Both parasitoid species were identified through scanning electron microscope (SEM) micrographs of the male genital capsules [31], and rDNA ITS2 sequences were analyzed for molecular identification, according to the methods described by Stouthamer et al. [32]. The GenBank accession numbers for *T. dendrolimi* and *T. ostriniae* were FR750279 and HE648326, respectively. Voucher specimens were kept at the Institute of Biological Control, Jilin Agricultural University, China. Parasitoid colonies were reared on eggs of rice moth *Corcyra cephalonica* (Stainton) (Lepidoptera: Pyralidae), under laboratory conditions (25 ± 1 °C, 70 ± 5%, relative humidity (RH) 16:8 (L:D) photoperiod). To maintain the initial parasitization ability of these parasitoids, after continuous rearing for five generations on *C. cephalonica* eggs, *T. dendrolimi* and *T. ostriniae* colonies were reared for a generation on their native host ACB eggs.

### 2.2. Host

#### Ostrinia furnacalis

To obtain ACB host eggs for the experiment, a moth mass-rearing was developed at the Institute of Biological Control, Jilin Agricultural University, Changchun, China. ACB larvae were kept under laboratory conditions, using a climate chamber with 25 ± 1 °C, 60 ± 5% RH, and 16:8 (L:D) photoperiod. The artificial diet for rearing *O. furnacalis* larvae was composed by wheat bran 300 g, yeast 100 g, methyl 4-hydroxybenzoate 8 g, sorbic acid 8 g, ascorbic acid 8 g, linoleic acid 50 µL, sucrose 28 g, agar 30 g, and water 1500 mL. The artificial diet was provided to the ACB larvae in plastic containers (23 cm × 23 cm × 5 cm). After pupation, the insects were collected and placed in a mesh cage (35 cm × 35 cm × 35 cm). After the moths emerged, a 20% honey solution (*v*/*v*) was provided as food on a cotton wick [2]. A large piece (30 cm × 30 cm) of wax paper lined the inner walls of the cage, serving as oviposition substrate. The wax paper containing newly laid eggs was removed as required. A previous study [30] showed that the duration of egg development of *O. furnacalis* was 88.6 ± 5.8 h. In preliminary experiments, we observed that the parasitism rate of *T. dendrolimi* dramatically declined (>50%) when the age of *O. furnacalis* host eggs was older than 8 h. Collectively, we evaluated the parasitism rate of *T. dendrolimi* and *T. ostriniae* on 0–48-h-old ACB eggs to ensure accurate host-age preference. The egg masses on the wax papers were cut out with scissors and held in a climate chamber until they reached the age needed for the experiments described below (i.e., 0–4, 4–8, 8–12, 12–16, 16–24, 24–36, and 36–48 h old).

### 2.3. Impact of ACB Egg Age on the Biological Parameters of T. dendrolimi and T. ostriniae

No-choice test: To determine the effect of host age on parasitization, two parasitoids, *Trichogramma dendrolimi* and *T. ostriniae*, parasitizes ACB eggs of various ages (i.e., 0–4, 4–8, 8–12, 12–16, 16–24, 24–36, and 36–48 h) separately. The environmental conditions for the experiment were 25 ± 1 °C, 70 ± 5% RH, and 16:8 (L:D) photoperiod. A newly emerged (<12 h old) mated adult female of *T. dendrolimi* or *T. ostriniae* was introduced into a glass tube (10 × 3 cm, length × diameter) containing ACB egg cards (50–70 eggs card^−1^) of various ages, as described above. In each glass tube, a 20% honey solution (v/v) on a cotton wick was provided as food for adult parasitoids. After parasitizing for 24 h, the female was removed from each glass tube, and the parasitized egg cards of each age were transferred separately to an incubator chamber (25 ± 1 °C, 70 ± 5% RH 16:8 (L:D) photoperiod) to allow the parasitoid development. Five days later, the egg cards belonging to each treatment were examined under a stereoscopic microscope (LEICA S6E, Germany) and the number of parasitized eggs (i.e., characterized by a dark color) was recorded. The parasitized egg cards were then placed back in an incubator chamber until adult parasitoid emergence. The date of emergence and sex of each parasitoid species from each host age stage were recorded. The developmental time (i.e., time elapsed from egg parasitization to the adult emergence) of *T. dendrolimi* and *T. ostriniae* on each host age stage was noted. Each treatment was replicated 15 times.

Choice test: The ACB eggs of seven different stages (i.e., 0–4, 4–8, 8–12, 12–16, 16–24, 24–36 and 36–48 h), all randomly stapled on a paper strip, were offered to *T. dendrolimi* and *T. ostriniae* wasps separately. The environmental conditions for the experiment were 25 ± 1 °C, 70 ± 5% RH, and 16:8 (L:D) photoperiod. Each egg card of ACB was comprised of 50–70 eggs of the specific age group. A newly emerged (<12-h-old) mated adult female of *T. dendrolimi* or *T. ostriniae* was introduced into a glass tube (10 × 3 cm, length × diameter) containing various ages of ACB eggs at the same time. After parasitizing for 24 h, the female adults were removed from each glass tube, and the parasitized eggs of each age were transferred separately to an incubator chamber (25 ± 1 °C, 70 ± 5% RH 16:8 (L:D) photoperiod) to allow for parasitoid development. The other operation procedures were the same as in the no-choice test above. Each treatment was replicated 15 times.

### 2.4. Statistical Analysis

The number of differently aged ACB eggs parasitized by two *Trichogramma* species, the percentage of parasitoid offspring emergence, the developmental time, and the percentage of female progeny under a no-choice test were analyzed using two-way analysis of variance (ANOVA) with the host age (7) and parasitoid species (2) as factors. The means were separated using a Tukey’s HSD test. All data were subjected to normality and homoscedasticity tests (Shapiro–Wilk test and Levene’s test) before ANOVA. Prior to the ANOVA, data on the female progeny (%) and emerged *T. dendrolimi* and *T. ostriniae* parasitoids (%) were arcsine square-root-transformed to normalize variances. When the ANOVA revealed significant effects of the factors, means were separated by the Student’s *t*-test. In the choice test, the preference to differently aged ACB eggs parasitized by each *Trichogramma* species was analyzed using a non-parametric Friedman test. SAS statistical software package (SAS Institute, Cary, NC, USA) was used for all statistical analyses, and figures were plotted relying to OriginPro 2017 SR2.

## 3. Results

### 3.1. Impact of Host Age on T. dendrolimi and T. ostriniae Parasitization

**Figure 1:**
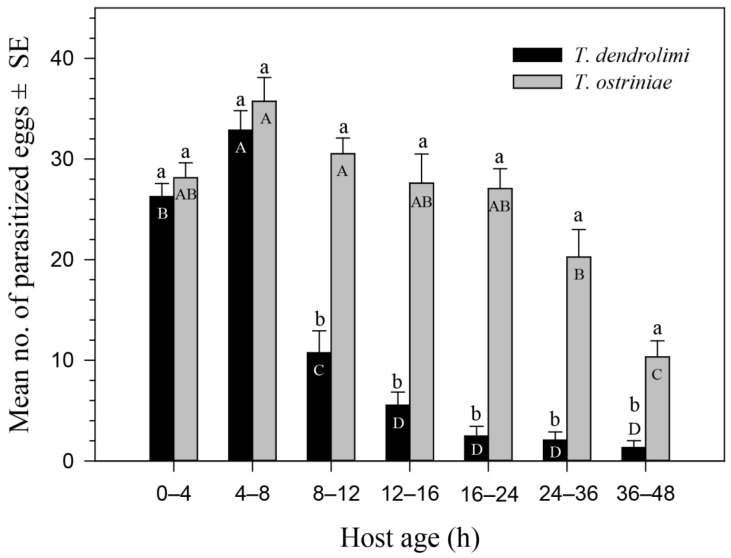
Suitability of the eggs of Asian corn borer (ACB) as host stages for the parasitization by *Trichogramma dendrolimi* and *Trichogramma ostriniae* under no-choice conditions. Mean No. of parasitized eggs ± SE are shown. Different upper-case letters on the same patterned bars indicate significant differences in parasitization of *T. dendrolimi* or *T. ostriniae* on ACB eggs with different ages, while different lower-case letters on the bars within a given group indicate significant differences in parasitization of *T. dendrolimi* and *T. ostriniae* on ACB eggs with the same age (Tukey’s HSD test, *p* < 0.05).

No-choice test: our results showed that the parasitoid species, host age, and interactions between these two variables had a significant impact on host parasitization (Table 1). As shown in Figure 1, the number of eggs parasitized by *T. dendrolimi* (*F*_6,98_ = 81.63, *p* < 0.0001) and *T. ostriniae* (*F*_6,98_ = 14.38, *p* < 0.0001) was significantly affected by the age of ACB eggs. *Trichogramma dendrolimi* parasitized the largest number of 4–8-h-old ACB eggs (32.9), followed by 0–4-h-old eggs (26.3) and 8–12-h-old eggs (10.7), while *T. dendrolimi* parasitized the lowest number of 12–48-h-old eggs (1.3–5.5), respectively. *Trichogramma ostriniae* showed no preference for ACB eggs that were 4–24 h old. As shown in Figure 1, *T. ostriniae* parasitized a significantly higher number of ACB eggs compared to *T. dendrolimi*, when various ages were tested.

**Figure 2:**
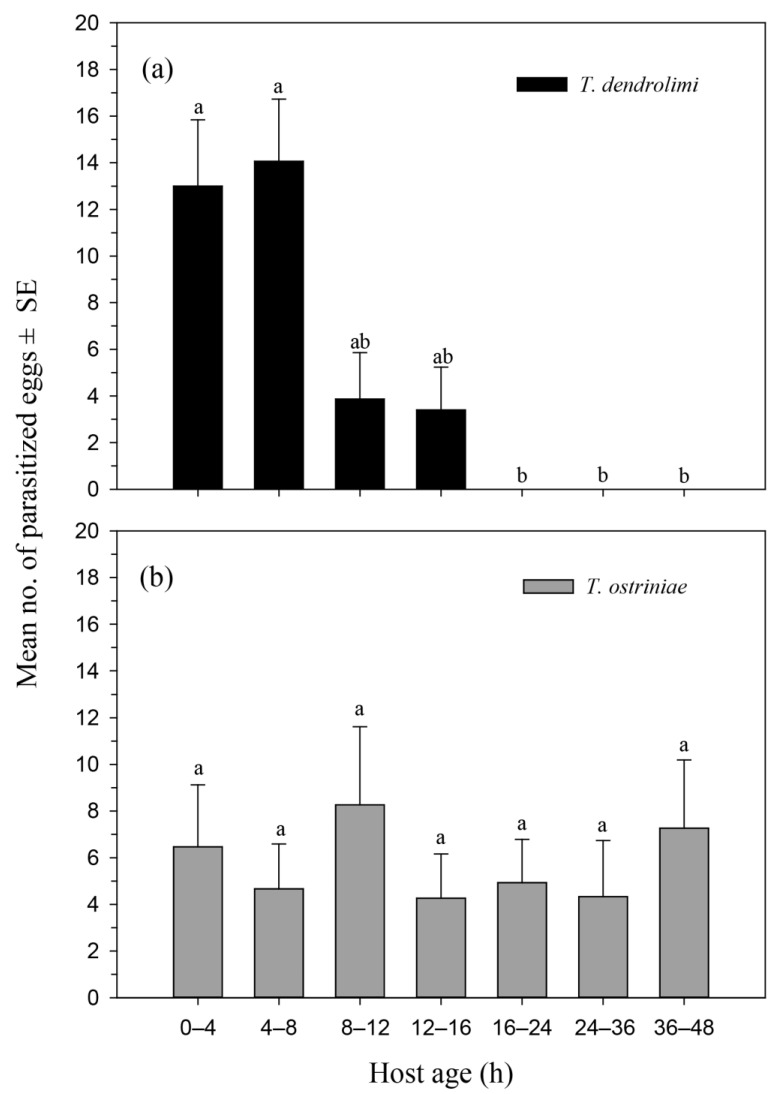
Suitability of the Asian corn borer (ACB) eggs as host stages for the parasitization by *Trichogramma dendrolimi* (**a**) and *Trichogramma ostriniae* (**b**) under choice conditions. Mean No. of parasitized eggs ± SE are shown. Different lower-case letters on the top of bars indicate significant differences in parasitization of *T. dendrolimi* and *T. ostriniae* on various ages ACB eggs (Friedman test, *p* < 0.05).

Choice test: the preference of differently aged ACB eggs parasitized by *T. dendrolimi* differed significantly while *T. ostriniae* species parasitized ACB eggs of all ages equally under choice conditions (Figure 2a,b). Results showed that the age of ACB eggs had significant effects on the parasitization ability of *T. dendrolimi* in choice conditions, where *T. dendrolimi* showed a strong parasitization preference to newly laid ACB eggs among various ages eggs (χ^2^ = 55.388, *df* = 6, *p* < 0.0001) (Figure 2a). *Trichogramma dendrolimi* parasitized significantly higher numbers of newly laid 4–8-h-old ACB eggs (14.06), followed by 0–4-h-old eggs (13.00). However, *T. dendrolimi* significantly parasitized a few numbers of ACB eggs that were 8–12 h old (3.86) and 12–16 h old (3.40). Particularly, *T. dendrolimi* did not parasitize 16–48-h-old ACB eggs in choice conditions (Figure 2a).

In contrast, the age of ACB eggs had no significant effect on the parasitization by *T. ostriniae* in the choice test; the parasitoids accepted all ACB egg-ages studied for parasitization (χ^2^ = 1.014, *df* = 6, *p* = 0.985) (Figure 2b). *Trichogramma ostriniae* parasitized ACB eggs of all ages equally.

### 3.2. Impact of Host Age on the Parasitoid Emergence, Development, and Female Sex Ratio

Parasitoid species and host age had no significant effects on the emergence, but their interactions affected parasitoid emergence (Table 1). No significant differences were observed in parasitoid emergence of *T. dendrolimi* (*F*_6,61_ = 1.85, *p* = 0.1052) and *T. ostriniae* (*F*_6,98_ = 1.17, *p* = 0.3277) from ACB eggs of various ages (Table 2). Overall, the emergence of both *T. dendrolimi* and *T. ostriniae* was higher for ACB eggs.

The tested *Trichogramma* species had a significant effect on the developmental time of the parasitoid, while the host age and their interactions did not show significant effects (Table 1). Similarly, there was no significant difference in developmental time of *T. dendrolimi* (*F*_6,61_ = 1.84, *p* = 0.1066) or *T. ostriniae* (*F*_6,97_ = 1.22, *p* = 0.3013) on ACB eggs of various ages (Table 3). However, significant differences were found in the developmental time between both species when compared at each tested age group of ACB eggs (Table 3). *Trichogramma ostriniae* developed significantly faster on each age group of ACB eggs than *T. dendrolimi*. As a general trend, *T. dendrolimi* showed the longest developmental time on ACB eggs among all treatments (Table 3).

Concerning the parasitoid female progeny, the tested *Trichogramma* species, the host age, and the interaction between these two factors had a significant effect (Table 1). A significant difference in the female progeny (%) of *T. dendrolimi* emerging from different ages of ACB eggs was noted, with a tendency for the female progeny (%) to decrease as the host egg age increased (*F*_6,61_ = 4.30, *p* = 0.0011) (Table 4). However, no significant differences in *T. ostriniae* female progeny (%) among different ages of ACB eggs were noted (*F*_6,97_ = 2.06, *p* = 0.0656) (Table 4). When parasitizing fresh ACB eggs (i.e., 0–4 h or 4–8 h old), *T. dendrolimi* and *T. ostriniae* had a similar percentage of female progeny. However, *T. dendrolimi* showed a significantly lower female progeny (%) on 8–12-h- to 36–48-h-old ACB eggs compared to *T. ostriniae* (Table 4). In all cases, the progeny of both parasitoid species was female biased.

## 4. Discussion

Selecting the most effective trichogrammatid species as biocontrol agents for ACB area-wide management is of high economic importance. Several studies reported that *O. furnacalis* is a poor host for *T. dendrolimi* [12,33]. Similarly, the parasitization rates of *T. dendrolimi* on ACB eggs were found to be low, ranging from 3.28 to 30% in various laboratory studies [30,34], compared to the high parasitization (100%) of *T. ostriniae* [34]. Furthermore, *T. ostriniae* was found to be the most common egg parasitoid of ACB in the field [21]. The parasitization rates of *T. dendrolimi* on ACB eggs were higher than 80% due to inundative releases (30,000 wasps/667 m^2^) [35]. However, further field surveys indicated that the post-release parasitization of *T. dendrolimi* on the subsequent generation of ACB decreased to less than 8%, unless it was supplemented by continued releases of the parasitoid, and *T. ostriniae* quickly became the dominant egg parasitoid [35,36,37]. All the above findings raise concerns about the effectiveness of *T. dendrolimi* against ACB in field trials, as well as why its initial successful parasitization rates at release time quickly fade in subsequent generations.

In this scenario, our results outlined that the host age is one of the key factors influencing the parasitoid’s host seeking behavior which characterizes this trophic interaction [38,39]. Herein, we evaluated how the age of ACB eggs can affect the key biological parameters in *T. dendrolim*i and *T. ostriniae*, to investigate a possible reason for the incompetency of *T. dendrolimi* on ACB eggs in the field. Our results showed that the age of host eggs (ACB) had a significant impact on *T. dendrolimi* and *T. ostriniae* parasitization activity under no-choice and choice conditions. *Trichogramma dendrolimi* parasitized a significantly higher number of 0–8-h-old ACB eggs, while a drastic decline in the number of eggs parasitized by *T. dendrolimi* was observed when the age of ACB eggs increased, i.e., >8-h-old eggs, in both no-choice and choice conditions. Furthermore, *T. dendrolimi* owns a strong choosiness behavior towards older ACB eggs, and is attracted towards freshly laid ACB eggs. The possible explanation for the high parasitism preference of *T. dendrolimi* towards freshly laid eggs and choosiness behavior towards older ACB eggs could be partly linked to the chorion structure and volatile semiochemicals produced by ACB eggs of different ages. Indeed, it has been reported that the structure of the chorion, such as thickness, is important for the acceptance of the host eggs by *Trichogramma* parasitoids [40]. Our findings agree with earlier research [41], where the authors investigated the effect of age on the parasitization potential of six *Trichogramma* species, including *T. dendrolimi*, on the eggs of *Mythimna separata* Walker (Lepidoptera: Noctuidae). The authors also observed a high parasitization preference of *T. dendrolimi* for younger eggs of *M. separata*. Similarly, three tested *Trichogramma* species, including *T. dendrolimi*, showed a tendency to parasitize younger *C. suppressalis* eggs, both in choice and no-choice conditions [19]. The low parasitization rates of *T. dendrolimi* on ACB eggs may be due to the low toxicity of *T. dendrolimi* female venom against ACB eggs [42]. The parasitization rate of *Trichogramma* spp. can be affected by the age of the host egg [43]. Our results showed that ACB eggs, aged 0–8 h, were suitable host eggs under both no-choice and choice conditions. These findings indicated that ACB egg masses offer a short window of opportunity for *T. dendrolimi* to parasitize. So, to achieve high parasitization rates, *T. dendrolimi* should find ACB eggs soon after they are laid. Based on these observations, it is strongly recommended to release *T. dendrolimi* mixed development instars in the field (i.e., both larvae and pupae) instead of uniformly aged individuals, as well as to perform inundative releases throughout the oviposition period of ACB, for successful biological control programs. Certainly, the biocontrol strategy releasing *T. dendrolimi* against ACB should be tested in this field in the future.

Furthermore, our results pointed out that *T. ostriniae* can accept ACB eggs of various ages under no-choice and choice conditions, and could parasitize a similar number of ACB eggs between those aged 0–4 h and 16–24 h. These results confirm those by Iqbal et al. [2], who did not detect significant differences in the number of *T. ostriniae*-parasitized ACB eggs between those that were 12 h old and 24 h old. Our results are also in accordance with [44], who found no variations in the parasitization of *T. ostriniae* species between 1-day-old and 4-days-old host eggs of soybean pod borer *Leguminivora glycinivorella* (Matsumura) (Lepidoptera: Tortricidae), despite using a different host from ACB. *Trichogramma* *ostriniae* parasitized significantly more 4–12-h-old host eggs than 24–48-h-old eggs under no-choice conditions, whereas under choice conditions, this difference was practically absent. It could be explained that *T. ostriniae* females do not choose the age of *O. furnacalis* eggs during parasitism, as reported earlier [2]. In the choice test, 7 *O. furnacalis* egg ages were simultaneously available for *T. ostrinae* to parasitize, so it may be possible that the female parasitoid randomly selected the age groups. Since host eggs of various ages are commonly found in the field, the acceptance of ACB eggs in a wider age range would be beneficial for the reproductive success of *T. ostriniae*.

In general, the evaluation of key biological parameters, including parasitization ability, parasitoid emergence, female progeny, and developmental duration on target host eggs, typically constitutes the basis for using *Trichogramma* parasitoids in biocontrol programs [2,45,46,47,48,49]. According to our studies, the parasitization ability of *T. dendrolimi* and *T. ostriniae* on ACB eggs of different ages varied, but *T. ostriniae* accepted host eggs of all ages.

In a typical biological control program, egg parasitoids should be able to parasitize a large number of eggs and develop female-biased offspring [50]. *Trichogramma ostriniae* parasitization led to comparable abundance of newly emerged adult females from different ages of ACB eggs, while *T. dendrolimi* led to different abundance of newly emerged adult females from ACB eggs of different ages. However, both species developed a female-biased progeny on all ages of ACB eggs, according also to a recent study [2]. Furthermore, our results showed no variations in *T. ostriniae* developmental time on ACB eggs of various ages, while some differences in the developmental time of *T. dendrolimi* on the same host were noted. In comparison to *T. dendrolimi*, *T. ostriniae* developed faster on ACB eggs. This suggests that *T. ostriniae* outperforms *T. dendrolimi* in parasitizing ACB eggs of various ages.

The practical advantages of producing *Trichogramma* parasitoids using host eggs from the Chinese oak silkworm, *Antheraea pernyi* (Guérin-Méneville) (Lepidoptera: Saturniidae), have been recently reported, including their high parasitization rate, easy storage, and transportation [51]. In comparison to production on small host eggs, the cost of producing *Trichogramma* using *A. pernyi* eggs showed a significant eight-fold decrease [12,52]. *Trichogramma dendrolimi* and *T. ostriniae* have been found to have several advantages for a successful management of ACB moths. Monoparasitism allows *T. dendrolimi* to be mass-produced on eggs of its factitious host *A. pernyi* [53]. On the other hand, *T. ostriniae* can be mass-reared efficiently by multiparasitism with other *A. pernyi*-capable species [12,54]. The efficacy and biological traits of *T. dendrolimi* and *T. ostriniae* species reared with *A. pernyi* on the target ACB have also been thoroughly investigated [2,30]. Overall, considering the results of our study, we highlight that *T. dendrolimi* prefer to parasitize younger ACB eggs, especially 0–8-h-old eggs, while *T. ostriniae* is able to exploit a wide range of ACB egg ages. We can, therefore, recommend releasing *A. pernyi*-reared *T. ostriniae* species for the successful biological control of ACB.

## 5. Conclusions

We evaluated the reason for incompetency of *T. dendrolimi* species to parasitize ACB eggs in comparison with *T. ostriniae* species. The study concluded that the age of ACB eggs was a key factor that significantly affected the parasitism capability of *T. dendrolimi* species in comparison to *T. ostriniae* species, in both choice and no-choice conditions. *Trichogramma dendrolimi* showed significant high parasitism preference to younger ACB eggs (0–8 h old), while *T. ostriniae* parasitized ACB eggs of all ages in significantly high numbers. Overall, these findings provide a groundwork for the selection and augmentation of potential biocontrol agents against ACB in corn fields.

## Figures and Tables

**Table 1:** Results of two-way ANOVA testing the effects of parasitoid species (PS), host ages (HA), and their interactions on the performance of *Trichogramma dendrolimi* and *Trichogramma ostriniae* in terms of parasitization, parasitoid emergence, development, and female progeny.

Parameter	Variance Source	*df*	*F*	*P*
Parasitization	PS	1	207.68	<0.0001
	HA	6	56.22	<0.0001
	PS × HA	6	13.13	<0.0001
	Error	196		
Percentage of emergence	PS	1	0.13	0.7164
	HA	6	0.69	0.6596
	PS × HA	6	2.88	0.0122
	Error	158		
Developmental time	PS	1	204.75	<0.0001
	HA	6	1.84	0.0944
	PS × HA	6	1.48	0.1896
	Error	158		
Percentage of female progeny	PS	1	25.92	<0.0001
	HA	6	5.72	<0.0001
	PS × HA	6	3.25	0.0048
	Error	158		

**Table 2:** Emergence (%) of *Trichogramma dendrolimi* (TD) and *Trichogramma ostriniae* (TO) on Asian corn borer (ACB) eggs of different ages.

Parameter		Host Age (h)						
		0–4	4–8	8–12	12–16	16–24	24–36	36–48
Emergence (%)	TD	90.2 ± 2.0 Ab	92.7 ± 1.2 Aab	96.7 ± 1.5 Aa	95.9 ± 2.4 Aa	97.2 ± 1.8 Aa	96.7 ± 3.3 Aa	91.4 ± 4.2 Aab
	TO	95.6 ± 0.8 Aa	94.2 ± 1.3 Aa	93.8 ± 1.1 Aab	92.5 ± 1.6 Aa	92.1 ± 1.0 Aab	94.5 ± 1.9 Aab	95.6 ± 1.2 Aa
		*t* = 2.4689	*t* = 0.8526	*t* = 1.5422	*t* = 1.2648	*t* = 2.6094	*t* = 0.5992	*t* = 1.3755
		*df* = 28	*df* = 28	*df* = 24	*df* = 23	*df* = 19	*df* = 19	*df* = 17
		*p* = 0.0199	*p* = 0.4011	*p* = 0.1361	*p* = 0.2186	*p* = 0.0172	*p* = 0.5561	*p* = 0.1868

For each parameter, means ± SE are shown. Each value followed by different upper-case letters in the row indicate significant differences of *T. dendrolimi* or *T. ostriniae* on ACB eggs with different ages, while different lower-case letters in column indicate significant differences of *T. dendrolimi* and *T. ostriniae* on ACB eggs with the same age (Student’s *t*-test, *p* < 0.05).

**Table 3:** Developmental time of *Trichogramma dendrolimi* (TD) and *Trichogramma ostriniae* (TO) on Asian corn borer (ACB) eggs of different ages.

Parameter		Host Age (h)						
		0–4	4–8	8–12	12–16	16–24	24–36	36–48
Developmental time (days)	TD	9.9 ± 0.0 Aa	9.8 ± 0.1 Aa	9.9 ± 0.1 Aa	9.9 ± 0.1 Aa	9.9 ± 0.1 Aa	10.0 ± 0.2 Aa	10.1 ± 0.1 Aa
	TO	9.4 ± 0.1 Ac	9.4 ± 0.1 Ab	9.4 ± 0.1 Ab	9.5 ± 0.1 Ab	9.3 ± 0.1 Ab	9.3 ± 0.0 Ab	9.5 ± 0.1 Ab
		*t* = 6.4047	*t* = 4.7280	*t* = 6.1053	*t* = 3.8620	*t* = 5.7076	*t* = 6.3188	*t* = 5.1907
		*df* = 28	*df* = 28	*df* = 24	*df* = 23	*df* = 19	*df* = 19	*df* = 17
		*p* < 0.0001	*p* = 0.0001	*p* < 0.0001	*p* = 0.0008	*p* < 0.0001	*p* < 0.0001	*p* = 0.0001

For each parameter, means ± SE are shown. Each value followed by different upper-case letters in the row indicate significant differences of *T. dendrolimi* or *T. ostriniae* on ACB eggs with different ages, while different lower-case letters in column indicate significant differences of *T. dendrolimi* and *T. ostriniae* on ACB eggs with the same age (Student’s *t*-test, *p* < 0.05).

**Table 4:** Female progeny (%) of *Trichogramma dendrolimi* (TD) and *Trichogramma ostriniae* (TO) on Asian corn borer (ACB) of different ages.

Parameter		Host Age (h)						
		0–4	4–8	8–12	12–16	16–24	24–36	36–48
Female progeny (%)	TD	85.1 ± 1.5 ABa	85.5 ± 1.3 Aa	73.4 ± 4.0 ABb	70.4 ± 2.7 ABc	66.7 ± 5.0 Bb	69.1 ± 11.2 ABb	68.0 ± 9.7 ABc
	TO	84.2 ± 1.3 Aa	84.3 ± 1.3 Aa	83.6 ± 1.5 Aa	81.0 ± 1.0 Ab	84.3 ± 1.9 Aa	84.3 ± 1.4 Aa	78.5 ± 2.6 Abc
		*t* = 0.4967	*t* = 0.6459	*t* = 2.8376	*t* = 4.2540	*t* = 4.0440	*t* = 2.1370	*t* = 1.4907
		*df* = 28	*df* = 28	*df* = 24	*df* = 23	*df* = 19	*df* = 19	*df* = 17
		*p* = 0.6233	*p* = 0.5236	*p* = 0.0091	*p* = 0.0003	*p* = 0.0007	*p* = 0.0458	*p* = 0.1544

For each parameter, means ± SE are shown. Each value followed by different upper-case letters in the row indicate significant differences of *T. dendrolimi* or *T. ostriniae* on ACB eggs with different ages, while different lower-case letters in column indicate significant differences of *T. dendrolimi* and *T. ostriniae* on ACB eggs with the same age (Student’s *t*-test, *p* < 0.05).

## Data Availability

Not applicable.
